# Constrained coding for error mitigation in nanopore-based DNA data storage

**DOI:** 10.1038/s41598-025-08531-z

**Published:** 2025-09-30

**Authors:** Kallie Whritenour, Mete Civelek, Farzad Farnoud

**Affiliations:** 1 Computer Science, University of Virginia, Charlottesville, USA; 2 Biomedical Engineering, University of Virginia, Charlottesville, USA; 3 Electrical and Computer Engineering and Computer Science, University of Virginia, Charlottesville, USA

**Keywords:** Information technology, Computer science, DNA sequencing

## Abstract

DNA has been proposed as an alternative to magnetic and solid-state devices for storing digital data. In DNA data storage, writing data is performed through DNA synthesis, and reading is done via sequencing. Nanopore devices for sequencing DNA, like those produced by Oxford Nanopore Technologies, allow long reads and real-time sequencing but with lower accuracy compared to other sequencers, such as Illumina. To improve the reliability of data storage in DNA, we aim to combat the high error rate of nanopore sequencing using constrained coding. Certain aspects of the physical process underlying nanopore sequencing mean that some sequences are more prone to sequencing errors than others. We leverage this observation to design constrained codes using constrained de Bruijn graphs, along with a state-splitting encoder and a Viterbi-based decoder. We find that the overall performance of our novel coding system substantially improves upon the state-of-the-art DNN-based methods, reducing sequence-level errors by up to 6 times. We also visually demonstrate the performance of our approach through the simulated recovery of an image encoded and decoded using our method.

## Introduction

Recently, DNA synthesis and sequencing have become increasingly more reliable and affordable. Given these advances, DNA is emerging as a promising candidate for future data storage solutions. Offering high data density^1^, efficient methods of replication^2^, and a long storage life^3,4^, DNA shows compelling advantages over current data storage methods^5^. Research in DNA data storage aims to leverage these characteristics to develop robust and reliable storage systems. Despite these advantages over traditional storage media, both synthesis and sequencing of DNA molecules can be prohibitively expensive^1,6^. This work focuses on improving the accuracy of nanopore sequencing, a technology that is both portable and comparatively inexpensive but suffers from a higher sequencing error rate^7^.

Many recent works in the field of DNA data storage have explored applications of error-correction techniques. Notable examples include the application of Reed-Solomon codes^8^ and Fountain Codes^9^ to address erasure errors. Constrained codes have also been investigated to avoid sequences more likely to produce basecalling errors, such as homopolymers and tandem repeats^9–11^. These approaches have also been combined into concatenated codes to apply both coding constraints and Reed-Solomon error correction as the outer code^12^. Other works have studied encoding methods for unique challenges and opportunities arising in the DNA data storage channel, such as combinatorial encoding methods to take advantage of the inherent redundancy in the synthesis process^13^ and identifying maximum information rate and appropriate encoding methods to address the unorderedness of DNA fragments^14^.

The current paper focuses on the reliability of nanopore sequencing^15^, a relatively inexpensive technology developed by Oxford Nanopore Technologies (ONT) that can potentially make data storage in DNA more cost-effective. To leverage this technology for DNA data storage, sources of error specific to nanopore sequencing must be handled appropriately, through error mitigation and correction. Examples of efforts in this direction include the use of convolutional codes to create a constrained set of codewords that are then decoded using trained weights from the ONT RNN basecaller’s final layer in conjunction with the Viterbi algorithm^16^. Researchers have also utilized a greedy approach to construct a set of short DNA sequences (tags) such that the electrical current signals, produced by nanopore sequencing, for any two are sufficiently different, thereby facilitating the identification of the short sequences^17^. This approach can be used for tagging and identifying longer sequences. However, it is not practical for DNA data storage encoding as there is no computationally feasible encoding or decoding algorithm. Nanopore sequencing’s inability to accurately handle homopolymer repeats has been addressed by introducing a run-length-limited (RLL) code^10^.

**Fig. 1:**
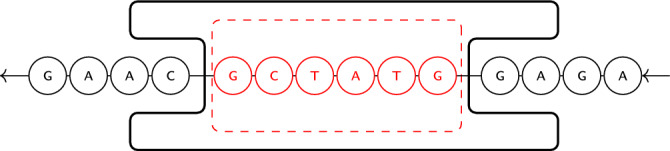
A DNA molecule passing through a nanopore. The *k*-mer in the pore is ${\textsf{G}}{\textsf{C}}{\textsf{T}}{\textsf{A}}{\textsf{T}}{\textsf{G}}$ and the next *k*-mer to enter the pore will be ${\textsf{C}}{\textsf{T}}{\textsf{A}}{\textsf{T}}{\textsf{G}}{\textsf{G}}$. The arrows indicate the direction of movement of the DNA molecule.

**Fig. 2:**
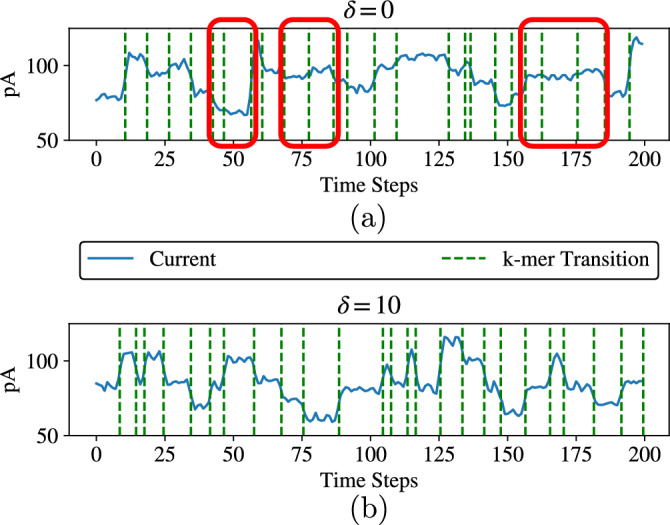
Example nanopore signals for (**a**) an unconstrained sequence ($\delta = 0$) and (**b**) a constrained sequence ($\delta ={10}\,\text {pA}$). Vertical dashed lines indicate transitions between k-mers. Events with hard-to-distinguish transitions are highlighted with red boxes for $\delta =0\,\text {pA}$. Signals are simulated by Deep Simulator.

In this work, we aim to enhance the reliability of nanopore sequencing by mitigating an important nanopore-specific source of error. To clearly describe the error source, we first provide an overview of the DNA data storage system studied in this paper. A data sequence ${{\varvec{x}}}\in \{0,1\}^n$, e.g., a document or an image, is encoded as a sequence ${{\varvec{y}}}\in \{{\textsf{A}},{\textsf{C}},{\textsf{G}},{\textsf{T}}\}^m$, which is then synthesized as a DNA molecule, storing the data. To read the data, the DNA molecule passes through a *nanopore* sequencer, as shown in Fig. 1. Specifically, the nanopore sequencer works by wrenching a single strand of DNA through a pore via motor proteins. At each point in time, a substring of *k* bases, called a *k*-mer, is inside this pore (in this work, we set $k=6$, in line with the r9.4.1 ONT nanopore). Then, the molecule is moved by one base, resulting in the next (overlapping) *k*-mer being placed inside the pore. Nanopore produces an electrical current signal whose value at a given time depends on the identity of the *k*-mer in the pore, but possibly also on other bases surrounding the nanopore. The signal is also affected by a random dwell time, i.e., how long the *k*-mer stays in the nanopore before the DNA molecule is moved ahead by one base. The current signal is sampled to produce a signal ${{\varvec{z}}}\in {\mathbb {R}}^M$, where *M*, the length of the signal, is affected by both the length of the DNA sequence and the dwell time of each *k*-mer that occurs during sequencing. An example of a (sampled) current signal is given in Fig. 2a, where vertical dashed lines indicate the instances in which the DNA molecule moves in the pore. The current signal is then decoded into a sequence ${{\hat{{{\varvec{y}}}}}}$ of bases, in a process referred to as *basecalling*, and is then decoded to a binary message $\hat{{\varvec{x}}}$.

One way to decode the current signal is to segment it into intervals, each corresponding to a *k*-mer. However, if two adjacent (overlapping) *k*-mers, e.g., $y_i,\dotsc ,y_{i+5}$ and $y_{i+1},\dotsc ,y_{i+6}$ produce similar current values, segmentation becomes challenging. Examples of *k*-mers with similar signals can be seen in Fig. 2a, highlighted using vertical rectangles (the vertical dashed lines represent true transition). Errors in segmentation may then lead to errors in the decoded signal.

In this paper, we propose constraining the DNA base sequence with the aim of ensuring that the electrical current values of each pair of adjacent *k*-mers are sufficiently different. We hypothesize that doing so will improve the performance of suitably designed segmentation-based decoders. It may also reduce errors in other decoders, e.g., Deep Neural Network (DNN) based decoders. We thus propose constrained codes, represented by a de Bruijn graph, where transitions are allowed only between *k*-mers whose expected electrical currents differ by more than a given threshold $\delta$. An example of the current signal for such a sequence is given in Fig. 2b, where it can be observed that transitions between *k*-mers with similar currents are almost entirely eliminated. For the proposed codes, we construct a state-splitting encoder and a Viterbi decoder. We show, using a state-of-the-art nanopore simulator, that this approach, compared to state-of-the-art DNN basecallers, reduces the error rate as measured by edit distance by almost a factor of 6.

Similar to other error control methods, our proposed constrained-coding approach achieves this reduction in error rate at the cost of the data rate, i.e., the number of bits of information that each base in a sequence carries on average, as discussed in Methods. As an alternative to preventing errors with a constrained code, one can add redundancy to the data using an error-correcting code and then correct the errors after retrieval. Our constrained codes are substantially more effective than the state-of-the-art error-correcting codes. In particular, we achieve a much higher data rate compared to using the most efficient codes for correcting insertions, deletions, and substitutions^18^, to eliminate the same number of errors. This comparison is further detailed in Results. Furthermore, for moderate threshold values, the performance of both state-of-the-art DNN-based decoders, Guppy and Dorado, also improve compared to unconstrained sequences. We also analyze how each type of error, namely insertions, deletions, and substitutions, changes as the threshold varies.

Finally, to show the performance of our proposed code within an end-to-end data storage system, an image was encoded into DNA sequences, passed through the simulated nanopore sequencer, and then decoded. The number of sequences needed to encode the image was dependent on the rate of the code and subsequently the $\delta$ value. We studied the prevalence of errors for different values of $\delta$ and read depths. We showed in particular that the image can be successfully retrieved with $\delta \ge {4}\,\text {pA}$ and read depth 30.

## The nanopore channel

An overview of the nanopore channel was presented in the previous section and a diagram shown in Fig. 1. In this section, we will provide further detail about nanopore sequencing, discuss simulators for the channel and their use in designing and evaluating the proposed codes, and provide a memoryless model used in decoding.

As stated, the DNA molecule passes through the pore such that at each point in time, there are *k* bases in the pore, where we set $k=6$. The movement is step-wise, where in each step, the molecule moves by one base. The duration that a given *k*-mer spends in the pore is called its *dwell time*, which is a random quantity. DNA moves through the nanopore at a speed of around 450 bases per second^19,20^, and so, on average each *k*-mer spends around 2.22 ms in the pore. The current signal produced by the DNA in the nanopore is sampled at 4000 Hz. Hence, on average, for each *k*-mer, we obtain around 8.89 signal values. Of course, due to the randomness of the dwell times, signal values are not deterministically associated with specific *k*-mers. Additionally, the signal values for a given *k*-mer are also noisy. For each of the $4^6=4096$ possible 6-mers, Oxford Nanopore Technologies (ONT) provides the mean and the variance of the current values^19,21^. The actual signal values, however, depend on the surrounding bases in a complicated manner^19^. All of these make identifying the sequence of bases comprising a DNA molecule from its nanopore current signal a challenging task.

### Simulation of channel

Encoding and decoding methods that we develop should ideally be evaluated through an experiment using nanopore sequencers. However, in DNA data storage, these tasks rely on synthesizing and sequencing a large number of DNA molecules, which is challenging and costly. As a surrogate for real-world experiments, faithful simulators of the channel can be used. As a first approximation, the mean current values and variances provided by ONT^21^ can be used to generate a simulated stochastic current signal for a given DNA sequence. However, as the current signal depends not only on the bases inside the pore but also on other bases in the sequence, such an approach suffers from lack of accuracy, especially for evaluating a proposed system.

ONT provides a Recurrent Neural Network (RNN) based simulator for the nanopore channel, called *Scrappie* (available through a developer license). DeepSimulator^19^ is another deep learning-based nanopore current simulator. We use DeepSimulator’s context-dependent tool to simulate the nanopore channel as its noise levels have been shown to be closer to true nanopore current variation and is more up-to-date with emerging nanopore technologies^19^.

Given a DNA sequence, DeepSimulator first produces a single value for each *k*-mer using a Bidirectional Long Short-Term Memory (Bi-LSTM) RNN architecture. Then each value is repeated according to a certain distribution to simulate the random dwell times. The resulting signal is then passed through a low-pass filter with a cut-off of 950 Hz to eliminate the high frequencies present in square waves. Finally, independent Gaussian noise (with a default variance of $1\,\text {pA}$) is added to each signal value. When using DeepSimulator in this work, sequences are padded at the front and back to avoid errors related to simulating the current signal without enough surrounding context.

## Methods

**Fig. 3:**
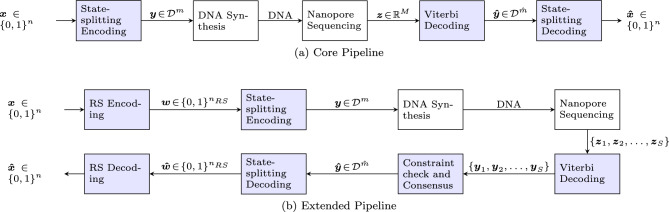
Pipelines for the encoding and decoding of data using constrained codes. (**a**) the core pipeline (for a single read and without additional error correction): binary data ${{\varvec{x}}}$ is encoded to a sequence over the alphabet ${\mathcal {D}}=\{{\textsf{A}},{\textsf{C}},{\textsf{G}},{\textsf{T}}\}$, which is then synthesized as a DNA molecule. For retrieval, this molecule is sequenced using a nanopore sequencer, producing a digital current signal ${{\varvec{z}}}$ with (quantized) real values. Viterbi basecalling produces a DNA sequence ${{\hat{{{\varvec{y}}}}}}$, possibly different from ${{\varvec{y}}}$, which is then decoded using the State-splitting decoder to produce the recovered data ${{\hat{{{\varvec{x}}}}}}$. This pipeline is used to investigate the fundamental aspects of the system, as presented in “Results”, including Figs. 8 and 9. (**b**) Demonstrates the Extended Pipeline with multiple reads and a Reed-Solomon outer code to correct residual errors. Data ${{\varvec{x}}}$ is RS-encoded as ${{\varvec{w}}}$, from which multiple sequences ${{\varvec{y}}}$ are produced by the state-splitting encoder, each representing part of ${{\varvec{w}}}$. For simplicity, only a single sequence ${{\varvec{y}}}$ is shown after the encoder, as the process for each sequence is similar. For the DNA molecule representing ${{\varvec{y}}}$, the nanopore produces *S* signals, ${{\varvec{z}}}_1,\dotsc ,{{\varvec{z}}}_S$, which are decoded by the Viterbi decoder, producing *S* reads, ${{\varvec{y}}}_1,\dotsc ,{{\varvec{y}}}_S$. These reads are used to produce a consensus sequence that satisfies the constraints, which is then decoded. This pipeline, representing an end-to-end DNA data storage system, is used to store and retrieve an image, as shown in Fig. 10.

In this section, we will discuss the construction of the proposed constrained codes, as well as the encoding and decoding processes. An overview of the data storage system is shown in Fig 3. The figure shows both a Core Pipeline focused on the fundamental aspects of our approach and an Extended Pipeline to handle residual errors and multiple reads, both discussed below. We start by describing the constrained codes in more detail.

### Constrained codes for accurate segmentation

As discussed in the Introduction and demonstrated in Fig. 2a, a main challenge for the segmentation of the current signal, produced by nanopore, into intervals representing distinct *k*-mers is that two *k*-mers may have very similar current values. This creates difficulties in differentiating transitions between adjacent (overlapping) *k*-mers, potentially resulting in inaccurate basecalling. To avoid such cases, we propose a constrained code consisting of a set of permitted sequences such that the difference between electrical current values for any two adjacent *k*-mers is larger than a given value $\delta$. We also limit the length of allowable homopolymer repeats to a maximum of 3 consecutive identical bases, called the max-run constraint.

To enforce the minimum difference in current, we use de Bruijn graphs to represent allowable sequences, which have been used previously to model constrained codes for various applications^22,23^. Specifically, the vertices of our de Bruijn graph are strings of length *k*, i.e., *k*-mers. Consider a *k*-mer (vertex) ${{\varvec{u}}}={\textsf{B}}_1{\textsf{B}}_2\cdots {\textsf{B}}_k$ where ${\textsf{B}}_i\in \{{\textsf{A}},{\textsf{C}},{\textsf{G}},{\textsf{T}}\}$ for $i=1,\dotsc ,k$. There are 4 outgoing edges from ${{\varvec{u}}}$ to ${\textsf{B}}_2\cdots {\textsf{B}}_k{\textsf{A}}$, ${\textsf{B}}_2\cdots {\textsf{B}}_k{\textsf{C}}$, ${\textsf{B}}_2\cdots {\textsf{B}}_k{\textsf{G}}$, and ${\textsf{B}}_2\cdots {\textsf{B}}_k{\textsf{T}}$. Now, a path in the de Bruijn graph represents a sequence over $\{{\textsf{A}},{\textsf{C}},{\textsf{G}},{\textsf{T}}\}$. Specifically, for the path ${{\varvec{u}}}_1{{\varvec{u}}}_2\cdots {{\varvec{u}}}_m$ the sequence is the *k*-mer representing ${{\varvec{u}}}_1$ concatenated with the last elements of ${{\varvec{u}}}_2,\dotsc ,{{\varvec{u}}}_m$.

To enforce our constraint in this de Bruijn graph, we label the edge from ${{\varvec{u}}}$ to ${{\varvec{v}}}$ by the difference between the approximate currents of the corresponding *k*-mers, i.e., by $|\mu _{{{\varvec{u}}}}-\mu _{{{\varvec{v}}}}|$, where $\mu$ is the mean current, in picoamps (pA), for the given *k*-mer obtained from official statistics released by ONT^21^. Edges are pruned if the edge label is below a threshold $\delta$ or if it results in a homopolymer run of length $\ge 3$ (e.g., the edge ${\textsf{G}}{\textsf{C}}{\textsf{A}}{\textsf{T}}{\textsf{A}}{\textsf{A}}\rightarrow {\textsf{C}}{\textsf{A}}{\textsf{T}}{\textsf{A}}{\textsf{A}}{\textsf{A}}$). Thus, we ensure any path in our de Bruijn graph represents a sequence such that the estimated difference between current values of adjacent *k*-mers will meet our threshold. The set of all sequences of a certain length corresponding to a path in the graph is a constrained code, where each sequence is called a codeword. Each codeword can represent a specific message.

**Fig. 4:**
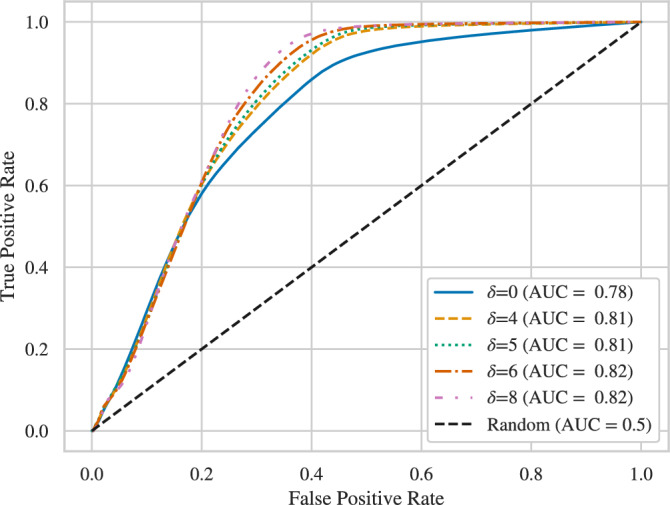
ROC curves for segmentation of current signals based on analyzing 1000 sequences of length 186 produced with default Deep Simulator parameters.

Here, $\delta$ is a tunable parameter. As $\delta$ increases and more edges are pruned, transitions between *k*-mers become easier to identify. To demonstrate this, we construct a simple method to segment the signal (we will later present a Viterbi decoder that simultaneously segments the signal and identifies the bases in each *k*-mer). Given the current signal ${{\varvec{z}}}$ and a timestep *t*, to determine whether there is a transition between $z_t$ and $z_{t+1}$, define $a = \frac{\sum _{i=t-w+1}^t z_{i}}{w},b= \frac{\sum _{i=t+1}^{t+w}z_i}{w}$ to be the averages of signal values in windows of length *w* before *t* (inclusive) and after *t* (exclusive). We choose $w=3$ as the dwell time is rarely less than 3. For a threshold $\delta$, if $|a-b|>\delta$, we declare a transition between adjacent *k*-mers. By varying $\delta$, one can control the trade-off between the false positive and false negative rates and obtain the Receiver Operating Characteristic (ROC) curves. These curves, along with the associated Area Under the Curve (AUC) values, are given in Fig. 4 for $\delta = {0, 4,5,6,8}$ pA. The figure shows that increasing $\delta$ also increases the performance of segmentation.

**Fig. 5:**
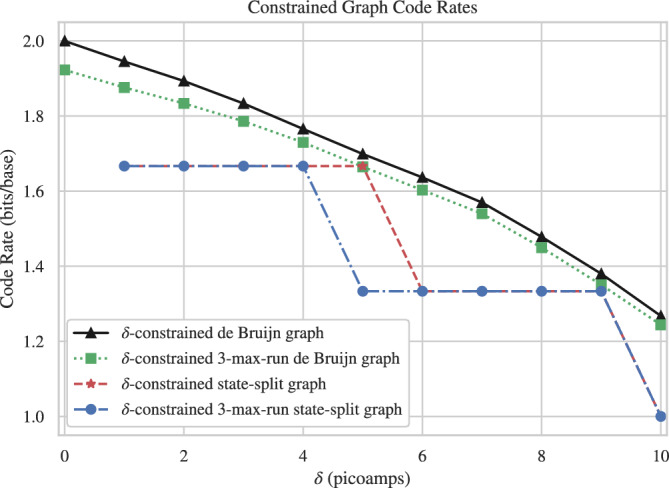
Code rate achieved for the de Bruijn graph constrained at different values of $\delta$, with and without the max-run constraint, for both the original de Bruijn graphs and the state-split graphs.

This increase in performance does come at a cost. Specifically, as $\delta$ increases, the number of possible sequences decreases, which in turn reduces the data rate, i.e., the number of bits of information that each base in a sequence represents on average, computed as the log of the number of permitted sequences divided by the number of bases in each sequence. For a de Bruijn graph *G*, the maximum achievable rate is denoted $\textsf{cap}(G)$, which can be calculated using the adjacency matrix of *G*^24^. For the proposed constrained code, the rate as a function of $\delta$ is shown in Fig. 5. The figure shows the rate for sequences satisfying the $\delta$ constraint only and also for sequences satisfying both the $\delta$ constraint and the homopolymer repeat constraint. (This figure also shows the actual code rate achieved by the state-splitting encoder discussed next.) We will later compare the trade-off achieved by our methods with other methods of increasing the reliability of nanopore sequencing.

### Encoding via the state-splitting algorithm

Data, represented as a sequence of bits, needs to be transformed into a sequence over the alphabet $\{{\textsf{A}},{\textsf{C}},{\textsf{G}},{\textsf{T}}\}$ so that it can be stored as a DNA molecule. This process is referred to as *encoding*. If all sequences are possible, a simple encoder that maps each pair of bits to an element of $\{{\textsf{A}},{\textsf{C}},{\textsf{G}},{\textsf{T}}\}$ suffices. But when there are constraints, and some DNA sequences are not permitted, other methods are needed.

In our approach, encoding was implemented using the state-splitting algorithm, which can construct finite-state encoders given a graph *G* representing a constrained code^25^. Our constrained code is determined by the parameter $\delta$ and is represented by the pruned de Bruijn graph. The algorithm works by successively splitting states, or vertices, of a power of the de Bruijn graph, until a minimum out-degree is reached. The resulting encoder structure can be changed through the algorithm’s input parameters, *p*, *q*, resulting in a graph with minimum out-degree of $2^p$, where the splitting is performed on $G^q$. The parameters *p*, *q* must satisfy $\frac{p}{q} \le \textsf{cap}(G)$. We choose *p*, *q* as close to the capacity of the input graph for each constraint separately, under the restriction that *q* must be small to reduce the complexity of the state-splitting algorithm, namely $q\le 6$. For nodes with $>2^p$ outgoing edges, extraneous edges were pruned in order of lowest edge labels. Then, encoding can then be done by assigning each edge a value from $\{0,1\}^p$. For the range of interest for $\delta$, the values of (*p*, *q*) are as follows: (10, 6) for $1\le \delta \le 5$ pA, and (8, 6) for $6\le \delta \le 9$ pA. The rates of the codes produced by the algorithm are given in Fig. 5, along with the capacities of the constrained graph, for different values of $\delta$.

Our encoding also includes padding the beginnings and ends of sequences. Nanopore sequencing signals simulated by deep neural networks can have errors at the beginning and end due to a lack of surrounding bases to form context when predicting the signal value. To mitigate simulation artifacts and to lessen the impact of simulation choices such as sequence length, we pad DNA sequences with 12 random bases in the front and 18 random bases at the end, obtained by following random paths on the constrained de Bruijn graph. These are recorded and removed after decoding. Front padding is removed by matching it to the expected pattern. If the resulting sequence is longer than the original sequence (with no padding), the end padding is trimmed to obtain the original sequence length.

### Decoding of constrained codes

In this section, we will present the decoding algorithm for the proposed constrained codes. To do so, we utilize a Viterbi decoder. We will start by presenting a hidden Markov model for the sampled current signal produced by nanopore in the next subsection. This model does not take the constraints on the sequences into account. The constraints represented by the de Bruijn graph are taken into account in the following subsection, where we introduce the Viterbi decoder.

**Fig. 6:**
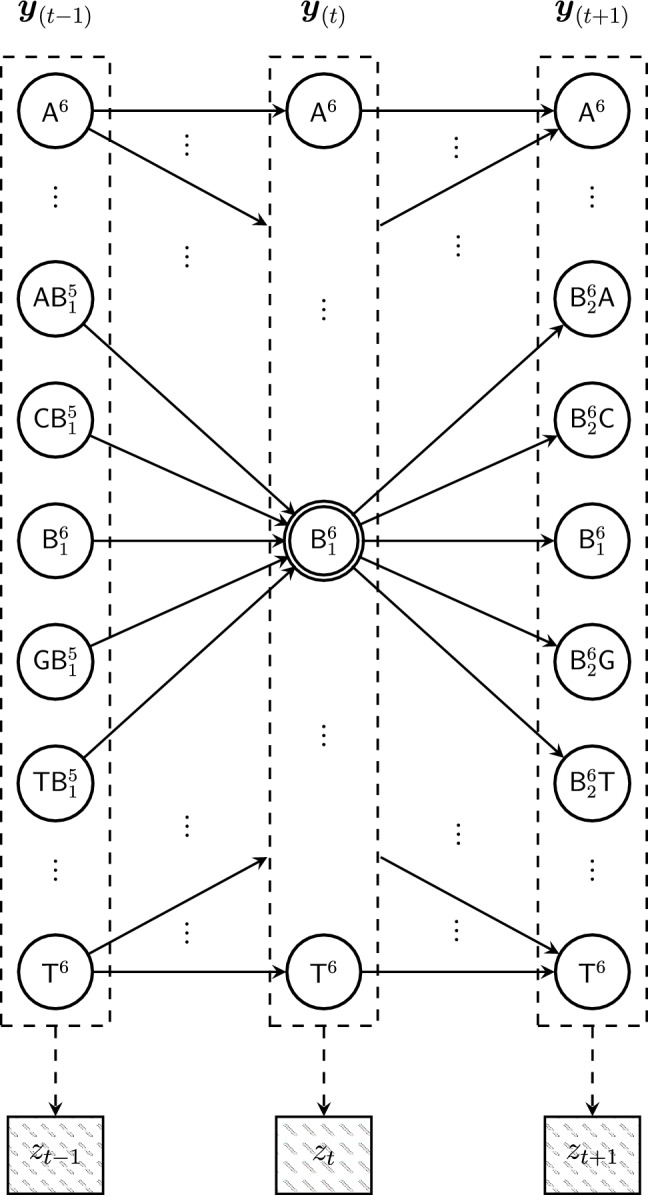
Trellis for the hidden Markov model representing the DNA sequence ${{\varvec{y}}}$ and the resulting current signal ${{\varvec{z}}}$. Each column represents a time step, and each circle node enclosed in a dashed box represents a possible hidden state. Observed emissions are the electrical signal values denoted by rectangle nodes. Here, ${\textsf{N}}^i$ means that the base ${\textsf{N}}\in \{{\textsf{A}},{\textsf{C}},{\textsf{G}},{\textsf{T}}\}$ is repeated *i* times, and ${\textsf{B}}_i^j = {\textsf{B}}_i\cdots {\textsf{B}}_j$. For a generic 6-mer ${\textsf{B}}_1\cdots {\textsf{B}}_6$ in the center of the diagram at time *t*, possible next states are given, including ${\textsf{B}}_1\cdots {\textsf{B}}_6$, which results from the DNA molecule not moving by one base through the nanopore.

#### Hidden Markov model for nanopore channel output

A hidden Markov model (HMM) for the nanopore current signal ${{\varvec{z}}}$ is presented in Fig. 6. As the nanopore channel is not memoryless, this model is not appropriate for evaluation. But it will be useful for constructing our Viterbi decoder in the next subsection. Consider a DNA molecule, representing a sequence ${{\varvec{y}}}$ of length *m* over the alphabet $\{{\textsf{A}},{\textsf{C}},{\textsf{G}},{\textsf{T}}\}$, passing through the nanopore, with $k=6$. At time *t*, the *k*-mer in the pore is denoted ${{\varvec{y}}}_{(t)}$, which is a substring of length 6 of ${{\varvec{y}}}$. Suppose ${{\varvec{y}}}_{(t)}={\textsf{B}}_1{\textsf{B}}_2\cdots {\textsf{B}}_6$ for ${\textsf{B}}_i\in \{{\textsf{A}},{\textsf{C}},{\textsf{G}},{\textsf{T}}\}$, shown at the center of Fig. 6, with label ${\textsf{B}}_1^6$. In the next time step, the DNA molecule will either remain in its position, leading to ${{\varvec{y}}}_{(t+1)}={{\varvec{y}}}_{(t)}$, or move one base through the pore, leading to ${{\varvec{y}}}_{(t+1)}={\textsf{B}}_2{\textsf{B}}_3\dotsc {\textsf{B}}_5 {\textsf{N}}$ for some ${\textsf{N}}\in \{{\textsf{A}},{\textsf{C}},{\textsf{G}},{\textsf{T}}\}$. We assume the molecule does not move with probability $\frac{1}{8}$ and when it moves by one base, all possibilities for ${\textsf{N}}$ have the same probability. So in this model, the dwell time is geometric, with an expected value of 8 timesteps. In each time step *t*, the nanopore produces an electrical current with value ${\varvec{z}_t}$, governed by a normally distributed emission whose mean and variance are determined by ${{\varvec{y}}}_{(t)}$. Specifically, $z_t\sim {\mathcal {N}}(\mu _{{{\varvec{y}}}_{(t)}}, \sigma ^2_{{{\varvec{y}}}_{(t)}})$, where $\mu _{{{\varvec{y}}}_{(t)}}, \sigma ^2_{{{\varvec{y}}}_{(t)}}$ are the ONT-reported mean and variance for the 6-mer ${{\varvec{y}}}_{(t)}$. We note that the dependence of the current values on only the current *k*-mer and geometric dwell times are not necessarily accurate but they will simplify the design of the decoder. Also, note that our evaluation relies on Deep Simulator, a neural network being capable of representing complex dependencies between the signal and the sequence, rather than the memoryless model described here.

#### Viterbi algorithm for basecalling

Using the hidden Markov channel model in Fig. 6, we can construct a Viterbi decoder. However, the model must be updated based on the constrained de Bruijn graph to eliminate edges between *k*-mers whose change in current values falls below the threshold $\delta$. This change requires us to also update the transition probabilities since some outgoing edges are eliminated. Let ${{\varvec{v}}}_1,{{\varvec{v}}}_2,\dotsc ,{{\varvec{v}}}_{4^k}$ represent the set of *k*-mers ordered lexicographically. Furthermore, let *A* be the $4^k\times 4^k$ adjacency matrix of the constrained de Bruijn graph. Transition probabilities were chosen to be uniform for all non-self transitions,

1 $$\begin{aligned} P(v_i \rightarrow v_j)&=\frac{\frac{1}{d}}{\sum _{j'=1}^{4^k} A_{ij'}} \end{aligned}$$

2 $$\begin{aligned} P(v_i \rightarrow v_i)&= 1 - \frac{1}{d} \end{aligned}$$

where *d* is the expected dwell time. With the updated hidden Markov model in hand, we implement the Viterbi decoder for segmentation and basecalling of the output signal of the nanopore device. We choose $k=6, d=8$, resulting in an average dwell time close to that of nanopore. Additionally, the Viterbi basecaller operates over the constrained de Bruijn graph prior to undergoing state-splitting. This is because the state-split graph has a much larger size, leading to infeasibly high computational complexity.

**Fig. 7:**
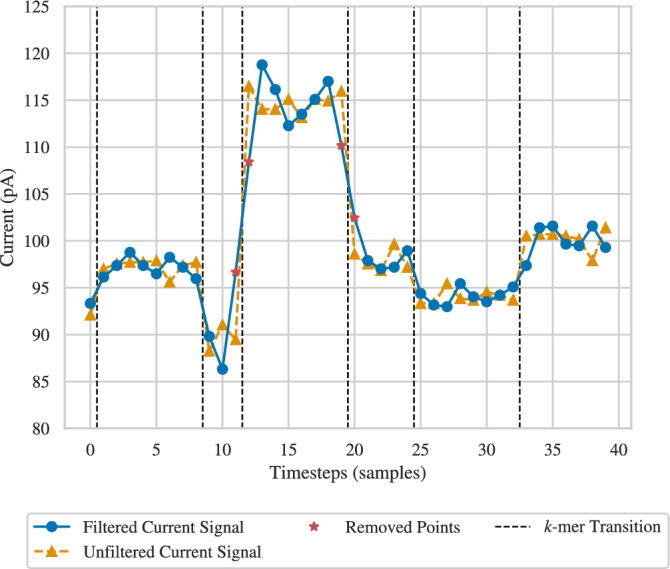
ISI occurs in nanopore signals due to the fact that the signal is band-limited. This is represented in Deep Simulator by applying a low-pass filter to a square-wave signal. The graph shows both the unfiltered signal (representing a signal with no ISI but with added noise) and the filtered signal with a cutoff of 950 Hz (the simulated output to which we have access). When the unfiltered signal changes rapidly, the values of the filtered signal also change rapidly but are inaccurate. The points indicated by $\star$ are removed from the filtered signal by the ISI-mitigation step.

#### Mitigating intersymbol interference

To further improve the reliability of the data storage system, we will now discuss combating a source of signal distortion, namely Intersymbol Interference (ISI). ISI is a phenomenon in communication and storage channels where the output of the channel at a given time is influenced not only by the current input but also by surrounding symbols (inputs), thus causing distortion. There are two main sources of ISI in nanopore sequencing. First, even though the *k*-mer inside the pore has the largest effect on the nanopore signal, the signal appears to depend in complex ways on other bases in the sequence. This source of ISI is difficult to characterize. The second, more manageable source results from the fact that the signal is naturally band-limited^19^, which Deep Simulator models via a low-pass filter. The dominant part of the effect of filtration can be seen in Fig. 7, where sharp rises at the transition between two *k*-mers are made smoother, leading to data points around the transition times affected not only by the current *k*-mer but also by the adjacent *k*-mer. This distortion may lead to decoding errors. In particular, these distorted data points may be interpreted as representing signal levels corresponding to a *k*-mer with a short dwell time, leading to an insertion. Alternatively, they may lead to the *k*-mer being misidentified, leading to a substitution error, or be interpreted as belonging to a neighboring *k*-mer, leading to a deletion.

To mitigate ISI resulting from the finite bandwidth of nanopore (represented in DeepSimulator via a low-pass filter), we can expand the Markov model for the channel (Fig. 6) to include states for transitions between two *k*-mers. However doing so would substantially add to the complexity of the Viterbi decoder. Instead, we opt for a simpler approach, namely, removing the points that appear to have been made inaccurate by ISI. Each 6-mer has a signal mean and standard deviation reported by ONT. Let ${{\hat{\sigma }}}$ denote the average of the standard deviations across all 6-mers. To remove data points potentially corrupted by ISI, we remove any point in the (filtered) signal that differs from the point before it and the point after it by at least $2\lfloor {\hat{\sigma }}\rfloor$, thus producing an ISI-mitigated signal.

In this way, data points that occur during a sharp rise or fall and differ from surrounding values by a larger amount than is expected from noise are viewed as points during transitions between *k*-mers. This approach can also be viewed as first performing segmentation on the signal and then removing the first and last points in each segment, which are most affected by ISI. We will then use the Viterbi algorithm described above to perform segmentation independently (and simultaneously with *k*-mer identification) on the ISI-mitigated signal. So, any errors in the ISI mitigation step do not necessarily translate into errors in decoding.

### Extended pipeline

While the Core Pipeline shown in Fig. 3a demonstrates the fundamental aspects of our approach, it needs to be extended to build an end-to-end DNA data storage system. The first reason is that while errors are reduced by the proposed error mitigation technique, they are not necessarily completely eliminated. The residual errors can be handled by an outer error-correcting code. Second, the Core Pipeline does not handle multiple reads. We now present the Extended Pipeline shown in Fig. 3b with the two aforementioned extensions. We note that other approaches for extending the Core Pipeline are possible, e.g., using other outer codes. For definiteness, we will consider storing an image shown in Fig. 10. However, the pipeline can be used for any type of digital data. (Experiments with image and text data^26^, are discussed in “Image storage and retrieval via Extended Pipeline” and Section B of the “Supplementary Appendix”.)

#### Outer RS code for residual error correction

Though our constrained inner code provides prevention for many errors, decoding information can still be difficult with the remaining errors, especially lost sequences and substitutions. Here, we employ a Reed-Solomon (RS) outer code over the field $\text {GF}(2^{12})$ and encode each image into a single codeword.

#### Handling multiple reads

In nanopore sequencing, preparation of the library to be sequenced typically results in multiple reads that are then used to find a consensus. In this work, we replicate this in our image decoding by simulating *S* reads of each sequence. Specifically, in Fig. 3b, for a given DNA molecule representing a sequence ${{\varvec{y}}}$ over $\{{\textsf{A}},{\textsf{C}},{\textsf{G}},{\textsf{T}}\}$, *S* electrical current signals, ${{\varvec{z}}}_1,\dotsc ,{{\varvec{z}}}_S$, are produced. Viterbi decoding for each sequence read is done over the constrained de Bruijn graph before splitting due to computational constraints. This allows some decoded sequences to violate constraints defined within the state-split graph. These sequences are discarded and the remaining sequences are used to determine a majority consensus. This consensus becomes our basecalled sequence that can now be decoded back into bits using the state-split graph.

## Results

**Fig. 8:**
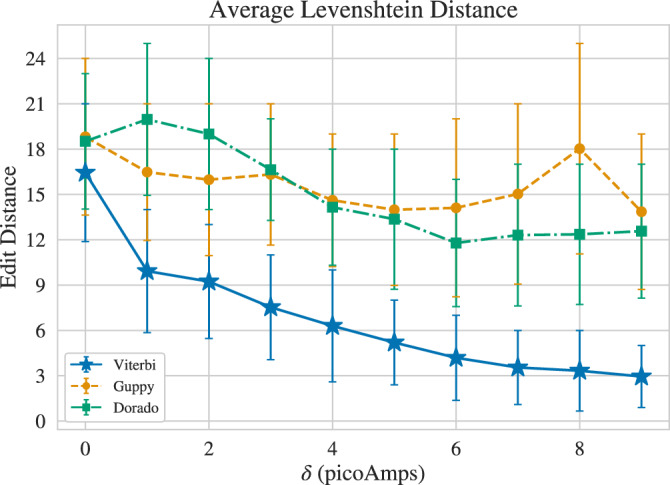
Basecalling performance on random unconstrained sequences ($\delta =0\,\text {pA}$) and constrained sequences produced by the state-split algorithm on de Bruijn graph ($\delta >0\,\text {pA}$), with 10000 sequences of length 186. Measured in average Levenshtein distance over all sequences for Viterbi Basecaller proposed by this work, the ONT Guppy Basecaller, and the ONT Dorado Basecaller. Error bars indicate 80th percentiles (20% of the probability mass is outside the range shown by the error bars).

**Fig. 9:**
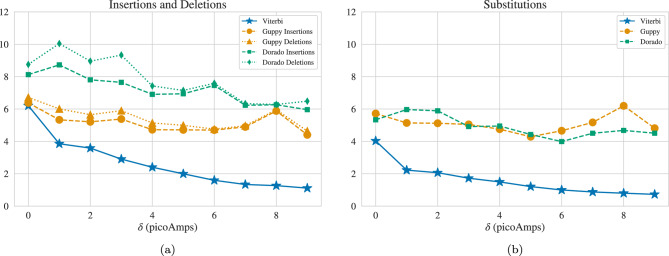
Basecalling performance on random ($\delta =0\,\text {pA}$) and constrained sequences ($\delta >0\,\text {pA}$), with 10,000 sequences of length 186. Average number of (**a**) insertions and deletions and (**b**) substitutions for the Viterbi Basecaller, proposed by this work, and the ONT Guppy and Dorado Basecallers.

**Fig. 10:**
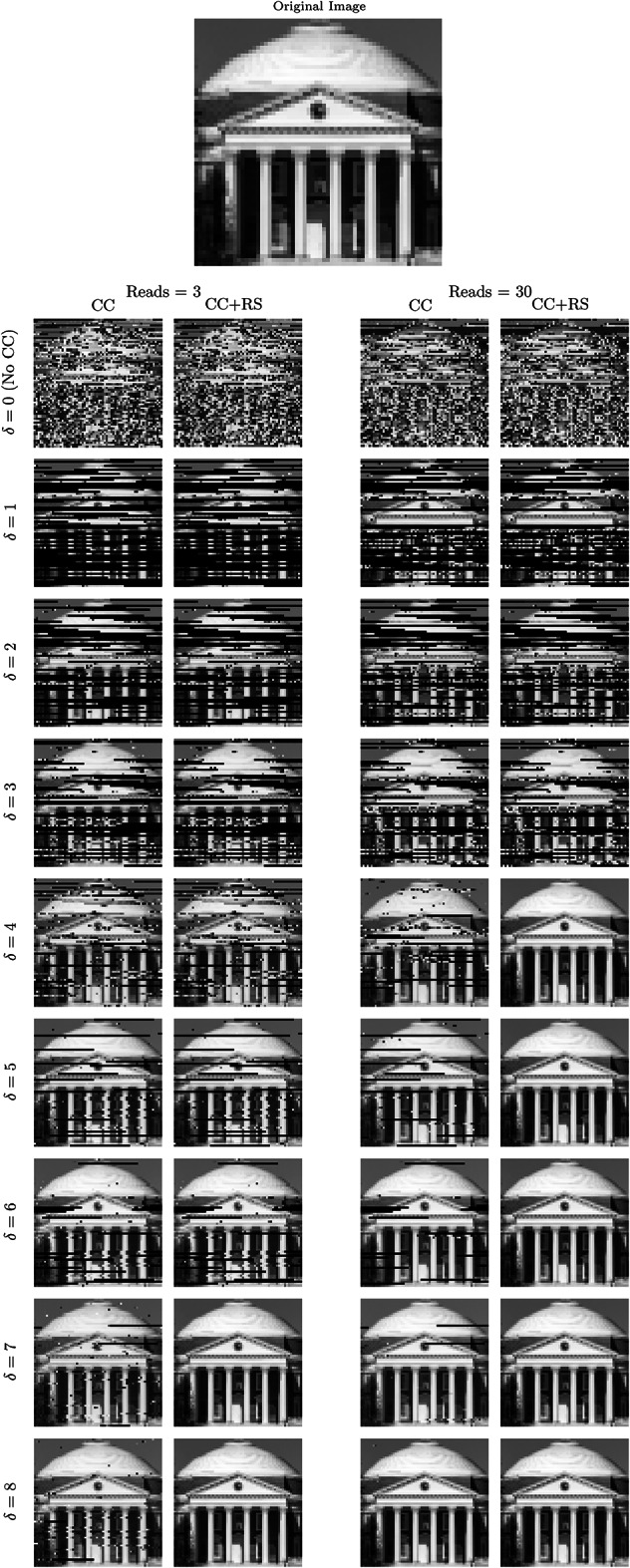
An image of the Rotunda at the University of Virginia, used as the original stored image (top left) and the decoded images for $\delta =0,\ldots ,8\,\text {pA}$ for 3 and 30 reads. The first row, with $\delta =0$, uses unconstrained sequences. Columns 1 and 3 (labeled CC) show the results using the proposed constrained codes, while columns 2 and 4 (labeled CC+RS) show the results where, additionally, a Reed-Solomon code is used to remove residual errors. Results for read depths 5, 10, and 20 are presented in Fig. 13 of the “Supplementary Appendix”.

**Table 1:** Bit-error rates for unconstrained sequences ($\delta =0$) and constrained codes with $\delta =1,\ldots ,8\,\text {pA}$ for 3 and 30 reads, with CC referring to using only the constrained code and CC+RS to also using RS codes.

$\delta$	Reads = 3		Reads = 30	
	CC	CC+RS	CC	CC+RS
0	0.379	0.379	0.469	0.469
1	0.310	0.310	0.302	0.302
2	0.242	0.242	0.257	0.257
3	0.213	0.213	0.203	0.203
4	0.140	0.140	0.082	0.000
5	0.115	0.115	0.070	0.000
6	0.110	0.110	0.048	0.000
7	0.072	0.000	0.014	0.000
8	0.060	0.000	0.001	0.000

To investigate the ability of our method to reduce errors in nanopore sequencing, we performed evaluations for both the Core Pipeline (Fig. 3a) and the Extended Pipeline (Fig. 3b). The evaluation of the Core Pipeline demonstrates the ability of our approach to reduce the number of edit errors and compares the performance to the state-of-the-art DNN-based basecalling methods, namely Guppy and Dorado developed by ONT^20,27^. Here, the performance is evaluated for single sequences using a single read. We then use the Extended Pipeline to simulate the storage and retrieval of an image, and study the impact of the choice of $\delta$ and the number of reads on the retrieval accuracy.

### Analysis of error rate for constrained codes

We first study the error rate of the proposed constrained code along with ISI-mitigated Viterbi decoding for different values of $\delta$ and compare it with the Guppy and Dorado basecallers. We measure the coding and basecalling (decoding) performance using Levenshtein distance, i.e. the number of edits (insertions, deletions, substitutions) required to transform the basecalled sequence to the true sequence. Specifically, the Levenshtein distance is measured between ${\varvec{y}}$ and $\hat{{\varvec{y}}}$ in Fig. 3a, where for evaluating the ONT basecallers, the Viterbi Decoding block is replaced by an ONT basecaller, either Guppy or Dorado. Here, the baseline is the performance of Guppy or Dorado for $\delta =0\,\text {pA}$, which corresponds to unconstrained sequences. For $\delta =0\,\text {pA}$, we produce random sequences over the alphabet $\{{\textsf{A}},{\textsf{C}},{\textsf{G}},{\textsf{T}}\}$, and for $\delta \in \{1,\dotsc , 9\}$ pA, constrained sequences are produced by the state-splitting encoder using random binary data. For each $\delta$, the Viterbi basecaller, the Guppy basecaller, and the Dorado basecaller were evaluated, in terms of both the total edit distance and individual error types over 10,000 sequences of length 186 bases. The results are presented in Figs. 8 and 9. Similar results for sequences of length 294 bases are presented in Supplementary Appendix A. We observe that there is no overlap between the Viterbi decoder’s 80th percentile intervals for constrained sequences ($\delta \ge 2\,\text {pA}$) and Guppy and Dorado’s 80th percentile intervals for *unconstrained* sequences, thus illustrating that our method can provide substantially lower error rates. For example, for $\delta =9\,\text {pA}$, the average edit distance for the Viterbi decoder (2.95) is at least 6 times lower than Guppy (18.81) and Dorado (22.21) on unconstrained sequences (obtained at the cost of approximately 0.7 bits/base in rate). Interestingly, the Viterbi decoder also outperforms both ONT decoders on unconstrained sequences. This difference can perhaps be explained by other factors, including the ISI-mitigation step. Nevertheless, the further improvement observed in the performance of the Viterbi decoder for increasing $\delta$ demonstrated the effectiveness of our constrained-coding approach.

We also observe that the constrained code leads to decreased average error for Guppy for all $\delta >0$ and for Dorado for $\delta >2\,\text {pA}$. In particular, for $\delta =5\,\text {pA}$, the average error for Guppy (13.99) is around 25% lower than its unconstrained baseline (18.81). Similarly, for $\delta =9\,\text {pA}$, the error rate for Dorado (16.95) is approximately 24% lower than its unconstrained baseline (22.21). These observations demonstrate that enforcing larger signal changes at transitions between *k*-mers may improve nanopore basecalling in general, even for basecallers without a specific mechanism to take advantage of it. While Dorado is the newer ONT basecaller, in our experiment it did not outperform Guppy. This is possibly because improvements in Dorado are not necessarily geared toward synthetic DNA, especially those with specific constraints. We also observed that in some cases, Dorado basecalls exhibited blocks of insertions or deletions at the beginning or end of the alignment. For Guppy, the results show that increasing $\delta$ beyond $6\,\text {pA}$ does not provide further gains, possibly because the sequence pool becomes too small, leading to a higher likelihood of tandem repeats. Seemingly, the errors resulting from such repeats cannot be compensated for by more clear transitions between *k*-mers. These observations suggest that a neural network-based decoder trained on the proposed constrained sequences may not only benefit from clear *k*-mer transitions but also address the aforementioned shortcomings of Guppy and Dorado. Such decoders can potentially have performance on par or better than Viterbi while being substantially faster.

The results also show that the proposed method outperforms the use of state-of-the-art error-correcting codes to eliminate errors. The best codes for correcting edit errors^18^ have redundancy at least $2t (2\log _2 n + \log _2 q)$ bits for correcting *t* edit errors in a *q*-ary sequence of length *n*. For $n=186$ and $q=4$, correcting each error would cost 17.3 symbols in redundancy. So correcting 12 edit errors would lead to redundancy as large as the sequence length, making the rate 0. The method proposed here, however, can prevent the same number of errors with $\delta = {3}\,\text {pA}$ at a rate of 1.67 bits/symbol. Similar statements can be made for other values of $\delta$.

In Fig. 9, the edit errors are broken down into insertions, deletions, and substitutions. For Viterbi, the number of insertion and deletion errors are equal because when we remove the padding as described in the Methods section, we obtain a sequence with the same length as the sequence without padding. Guppy and Dorado, however, occasionally produce sequences with so many deletions as to make it impossible to achieve the original length by removing padding. We observe that while all error types decrease for Viterbi as $\delta$ increases, insertion and deletion errors decrease by a larger margin. This may be expected as ensuring larger changes at the transitions between *k*-mers leads to fewer instances of misidentified transitions. Substitution errors decrease inversely with $\delta$ as well. This is likely because with the Viterbi basecaller, bases are not decoded individually and when a deletion or insertion occurs, the identity of the surrounding bases is decoded incorrectly to arrive at similar electrical current values. So minimizing segmentation errors will result in fewer substitutions.

### Image storage and retrieval via extended pipeline

We also evaluated the performance of the end-to-end Extended Pipeline in Fig. 3b via simulated image storage and retrieval for read depths $\{3, 30\}$, shown in Fig. 10. (Similar results for storing text data is presented in Supplementary Appendix B.) For this task, we encoded a 64$\times$64 pixel image, shown in Fig. 10, into sequences of length 186 (not including padding) with the encoding using 132, 158, 158, 158, 158, 198, 198, 198, 198 sequences for $\delta = 0, \ldots , 8$ pA, respectively ($\delta = 0\,\text {pA}$ corresponds to unconstrained sequences). The number of sequences changes because higher $\delta$s mean lower rates. Passing these sequences through the simulated nanopore channel, we decode the data to compare to the original image, as shown in Fig. 10. We also present the bit-error rate between the original and the decoded images in Table 1. This analysis corresponds to the distance between ${\varvec{x}}$ and $\hat{{\varvec{x}}}$ in Fig. 3b.

In Fig. 10 and Table 1, for both read depths and for retrieval with and without Reed-Solomon codes, we observe that increasing $\delta$ reduces the number of errors. In particular, for $\delta =0$, where the proposed constrained codes are not used, the image is almost completely lost. Increasing the number of reads and using RS outer codes improve the performance but only for larger values of $\delta$, where the constrained code ensures that each read is at least somewhat informative.

## Conclusion

We demonstrated that enforcing larger changes in the nanopore current signal at *k*-mer transitions using a constrained-coding approach, along with an appropriate decoder design, reduces the error rate of nanopore sequencing. Hence, our work can increase the reliability of DNA data storage pipelines that utilize nanopore sequencing. This work can be extended in several directions. First, an evaluation of the proposed method using experiments rather than simulation, as was done in this work, would provide a more accurate assessment. Additionally, we only addressed segmentation errors arising from sequencing. Hence, it is beneficial to consider other sources of errors, for example, those that arise during synthesis. Note that these errors may affect the validity of the constraints in the synthesized DNA and, thus, the segmentation performance. The encoding system may also be extended to address errors that occur in these sources, e.g., by enforcing GC-content. Additionally, we observe that the DNN-based decoders, Guppy and Dorado, also perform better for certain values of $\delta$, even though they are not aware of the constraints. Incorporating the constraints in this type of decoder could possibly outperform the Viterbi decoder as they are able to take channel memory into account. As nanopore technology advances, we also hope to extend the work to include new pore chemistries as well as any basecaller advancements that follow.

## Supplementary Information

Supplementary Information.

## Data Availability

The datasets generated and analyzed during the current study are available from the corresponding author upon request. Code used in this work can be found at https://github.com/kw5km/Nanopore-Constrained-Code
