# Supplementary material for: Constrained coding for error mitigation in nanopore-based DNA data storage
Source: Sci Rep. 2025 Sep 30;15:33899. doi: 10.1038/s41598-025-08531-z (PMC12484628; doi:10.1038/s41598-025-08531-z)
Supplement: Supplementary file 1 — Supplementary Information. [file 41598_2025_8531_MOESM1_ESM.pdf]

APPENDIX

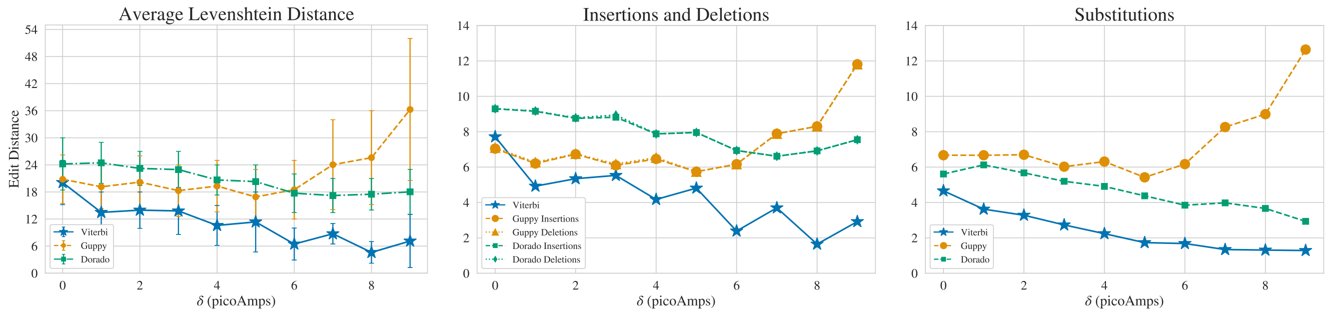

(a) (b) (c)

Figure 11: Basecalling performance on random ($\delta = 0\,\text{pA}$) and constrained sequences ($\delta > 0\,\text{pA}$), with 1000 sequences of length 294 bases (a), measured in average Levenshtein distance over all sequences for the Viterbi Basecaller proposed by this work, the ONT Basecallers, Guppy and Dorado. Error bars indicate 80th percentiles (20% of the probability mass is outside the range shown by the error bars). Average number of insertions and deletions (b) and substitutions (c) for the aforementioned basecallers.

Appendix A Performance of Longer Sequences

For completeness, we also investigate the performance of the proposed constrained code and Viterbi decoding compared with the ONT decoders, Guppy and Dorado, on a data set of 1000 sequences of 294 bases, similar to the analysis shown in 4.1, with the sequences produced in the same manner. Here, we measure the Levenshtein distance between the true sequence and the basecalled sequence for $\delta = 0, \dots, 9\,\text{pA}$, with $\delta = 0\,\text{pA}$ corresponding to unconstrained sequences. The total edit distance for this dataset is shown in Figure 11a.

We observe that the constrained code leads to decreased average error for the proposed Viterbi decoder for all values of $\delta > 0$. The same holds for Guppy for $\delta = \{1, 2, 3, 5, 6\}$ in Figure 11a, with errors increasing for larger δ, i.e., $\delta \geq 7\}$. We see the same U-shape trend in 8 as well, although $\delta = 9$ in 8 appears to be an outlier. For Dorado, we see general improvement for $\delta > 1$ in Figure 11a.

In Figure 11a, we see there is no overlap between the Viterbi decoder's 80th percentile intervals for constrained sequences with $\delta > 5$ and both Guppy and Dorado's 80th percentile intervals for unconstrained sequences, showing that our encoding offers improvement over the baseline.

Appendix B Encoding of Text Data

Performance was also evaluated for the end-to-end Extended Pipeline in 3b by simulating storage and retrieval of a text file for read depths $\{3, 30\}$. Results of the decoding are shown in Figure 12. Here, we use a 766 character length excerpt from the lecture by Richard Feynman "There's Plenty of Room at the Bottom" [10], with the plain text shown in Figure 12. We encode into sequences of length 186 (not including padding) using 16, 20, 20, 20, 20, 25, 25, 25, 25 sequences for $\delta = 0, \dots, 8\,\text{pA}$, respectively. We also employ a Reed-Solomon (RS) outer code as described in 3.4, except that we use a field of size $GF(2^{10})$ as our text data is smaller. Passing these sequences through the simulated nanopore channel, we decode the data to compare to the original text, as shown in Figure 12. Consistent with 10 and 1, Figure 12 shows that for both read depths and for retrieval with and without RS codes, increasing δ produces less erroneous decoding, with the data for 30 reads, $\delta = 8\,\text{pA}$, and CC+RS, recovered error-free (recovered data with 30 reads, $\delta = 8\,\text{pA}$, CC still contains errors).

```
This fact - that enormous amounts of information can be carried in an exceedingly small space - is, of course,
well known to the biologists, and resolves the mystery which existed before we understood all this clearly, of
how it could be that, in the tiniest cell, all of the information for the organization of a complex creature
such as ourselves can be stored. All this information - whether we have brown eyes, or whether we think at
all, or that in the embryo the jawbone should first develop with a little hole in the side so that later a
nerve can grow through it - all this information is contained in a very tiny fraction of the cell in the form
of long-chain DNA molecules in which approximately 50 atoms are used for one bit of information about the
cell.
```

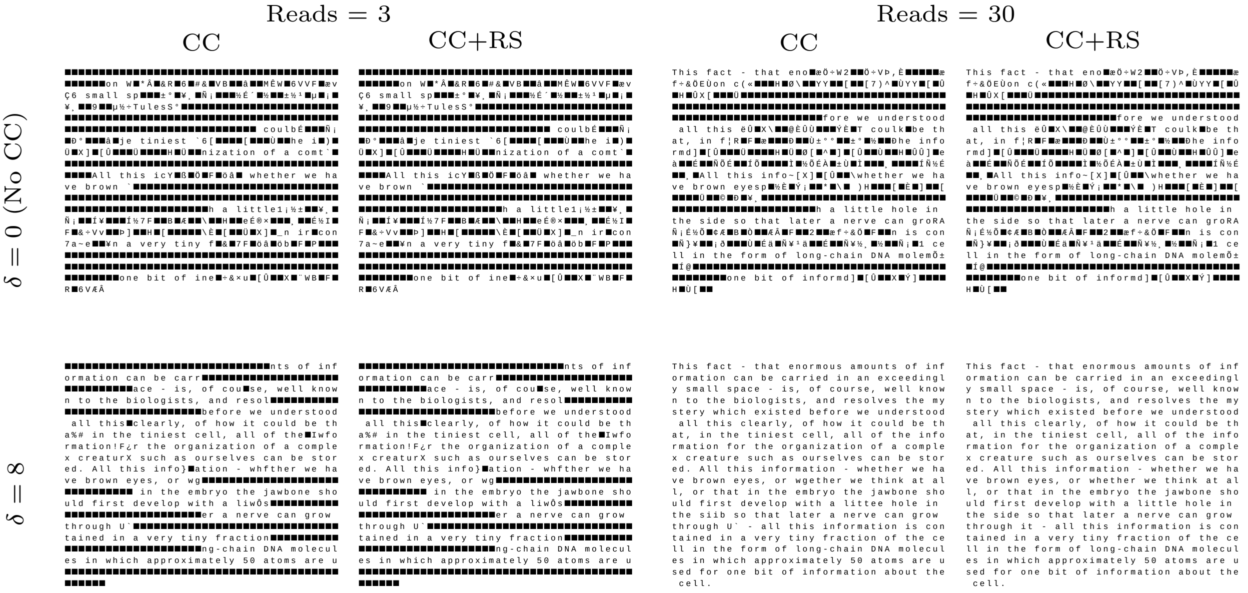

Figure 12: A transcript of an excerpt from Richard Feynman's lecture "There's Plenty of Room at the Bottom" [10], used as the original stored text (top) and the decoded text for $\delta = 0$ and $8\,\mathrm{pA}$, for 3 and 30 reads. The first row, with $\delta = 0$, uses unconstrained sequences. Columns 1 and 3 (labeled CC) show the results using the proposed constrained codes, while columns 2 and 4 (labeled CC+RS) show the results where, additionally, a Reed-Solomon code is used to remove residual errors. Errors resulting in erasures or unprintable characters are replaced with ■.

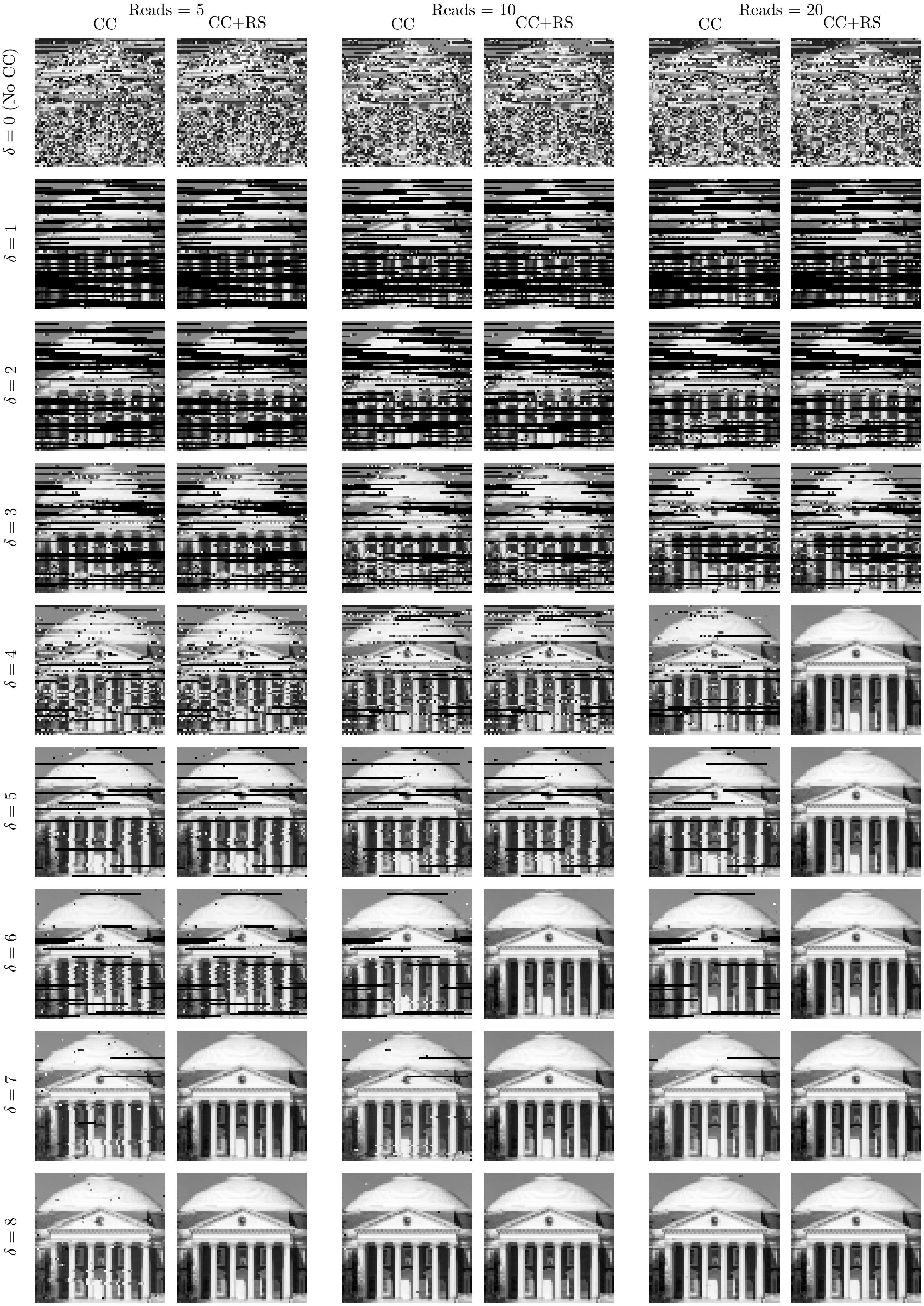

Figure 13: Image retrieval for read depths 5, 10, and 20, extending Fig. 10.
